# Axonal membrane stretch suppresses neuronal excitability by activating mechanosensitive K2P channels at the node of Ranvier

**DOI:** 10.1186/s13041-023-01000-6

**Published:** 2023-01-17

**Authors:** Hirosato Kanda, Koichi Noguchi, Yi Dai

**Affiliations:** 1 School of Pharmacy, Hyogo Medical University, Kobe, Hyogo 650-8530 Japan; 2 Laboratory of Basic Pain Research, Hyogo Medical University, Kobe, Hyogo 650-8530 Japan; 3 Department of Anatomy and Neuroscience, Hyogo Medical University, Nishinomiya, Hyogo 663-8501 Japan

**Keywords:** Node of Ranvier, Two-pore-domain potassium channels, Membrane stretch, Action potential, Single-channel activity

## Abstract

Saltatory conduction is the propagation of action potentials along myelinated nerves, which enables fast propagation through the node of Ranvier. Recently, we demonstrated that K2P channels, TWIK-related K^+^ channel-1 (TREK-1), and TWIK-related arachidonic acid-activated K + channel (TRAAK), are highly expressed in the mammalian node of Ranvier of sensory nerves and have an important role in action potential repolarization instead of voltage-gated K^+^ channels. TREK-1/TRAAK channels are activated by membrane depolarization as well as various stimuli, such as temperature, pH, arachidonic acid, and mechanical membrane stretch. Although membrane mechanical stretch has been suggested to modulate action potential conduction, how membrane stretching modulates intrinsic electrophysiological properties at the node of Ranvier remains unclear. In the present study, we examined the effects of membrane stretch on neuronal membranes at the node of Ranvier in rat sciatic nerves. The single-channel conductance was approximately 90 pS at 80 mV. Membrane stretch increased the single-channel event numbers and open probability in a pressure-dependent manner. Consistent with single-channel activity, intra-pipette positive pressure increased outward leak currents and decreased membrane excitability in a whole-cell configuration. Furthermore, blockage of TREK-1/TRAAK channels by Ba^2+^ reversed the changes in the intrinsic electrophysiological properties induced by intra-pipette pressure. These results indicate that the activation of mechanosensitive TREK-1/TRAAK channels may suppress neuronal excitability following axonal stretch. Our findings suggest that TREK-1/TRAAK channels may play an important role in the prevention of ectopic action potential discharge at the axon by intense mechanical nerve stretch under physiological conditions.

**Supplementary Information:**

The online version contains supplementary material available at 10.1186/s13041-023-01000-6.

## Introduction

The axon ensures stable action potential (AP) propagation from the axon initial segment to the axonal terminus [1]. While axons are generally considered reliable transmission cables, an AP can be initiated at the axon in an unusual condition in which intense pressure is directly applied to axons, or because of axonal injury, such as nerve compression syndrome and neuropathic pain [2, 3]. Recently, we and other have demonstrated that two tandem pore domain potassium (K^+^) channel (K2P channel) proteins, TREK-1 (TWIK-related K^+^ channel), and TRAAK (TWIK-related arachidonic acid-activated K^+^ channel) are highly expressed in the mammalian nodes of Ranvier (NRs) of the sensory nerve, and that AP repolarization at NRs relies on TREK-1/TRAAK channel activation [4, 5]. Interestingly, TREK-1/TRAAK are activated by membrane depolarization and by various stimuli, such as temperature, pH, arachidonic acid, and mechanical membrane stretch [5–8]. The suppression of TREK-1/TRAAK channel activities by cold temperature or pH delays AP repolarization and conduction velocity, and impairs the regeneration of high-frequency AP trains [4, 9]. Since TREK-1 and TRAAK are mechanosensitive K^+^ channels, axonal membrane stretching is likely to modulate the intrinsic electrophysiological properties of myelinated nerves. Liu et al. recently developed an experimental technique to apply an axonal mechanical stretch to ex vivo sciatic nerve preparation. Using these methods, they demonstrated that mechanical nerve stretch instantaneously mediates conduction delay in A-fibers [10]. Although membrane mechanical stretch has been suggested to modulate AP conduction, it is still unknown how membrane stretching modulates the excitability of axonal membranes at the NRs. In this study, we performed patch-clamp recording on ex vivo sciatic nerve preparations and examined the effects of membrane stretch on single-channel activity and intrinsic electrophysiological properties of the NRs.

## Results and discussion

**Fig. 1:**
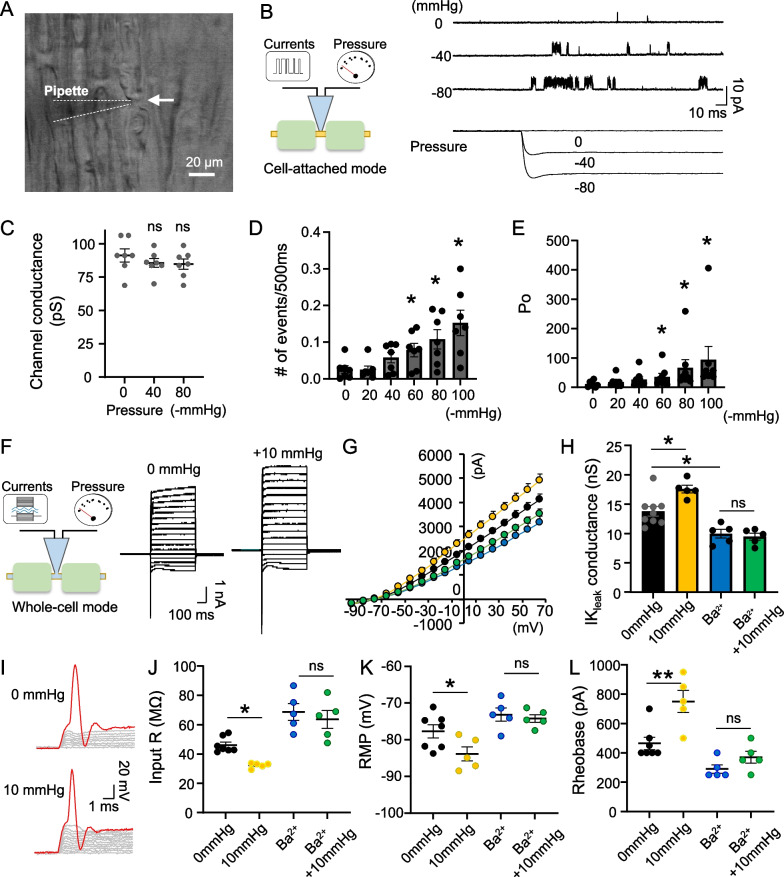
Effects of membrane stretch of neuronal membrane at the node of Ranvier (NR) of rat sensory nerve fiber. **A** Bright-field image showing patch-clamp recordings at an NR of Aβ-afferent nerve in ex vivo sciatic nerve preparation. **B** Left: recording setting of single-channel activity. Right: traces illustrating IK_leak_ single-channel currents at different intensities of membrane stretch on the nodal membrane. Summary of IK_leak_ single-channel conductance (**C**), single-channel events (**D**), and open probability (**E**) at different intensities of membrane stretch (n = 7). **F**–**H** Left: recording setting under the whole-cell patch-clamp. Right: Traces illustrating currents recorded at NRs by voltage steps (**F**), I–V curves of inactivating currents (**G**), and whole-cell conductance of IK_leak_ (**H**) following intra-pipette application of 0 mmHg (n = 9) and 10 mmHg pressure (n = 5), 0 mmHg pressure with Ba^2+^ (n = 5), and + 10 mmHg pressure with Ba^2+^ (n = 5). Traces illustrate typical action potentials recorded at NRs (**I**), input resistance (**J**), resting membrane potential (**K**), rheobase (**L**) following intra-pipette application of 0 mmHg (n = 7) and + 10 mmHg pressure (n = 5), 0 mmHg pressure with Ba^2+^ (n = 5), and + 10 mmHg pressure with Ba2^+^ (n = 5). Arrow indicates NR. Data represent mean ± SEM; ns, not significantly different; ∗p < 0.05, ∗∗p < 0.01, ∗∗∗p < 0.001, one-way ANOVA with Tukey’s multiple comparisons test

To determine the effect of membrane stretch at NRs of myelinated sensory nerves, we performed a pressure-clamped patch-clamp on intact NRs of rat sciatic nerve [11] (Fig. 1A). We first analyzed single-channel activities at the NRs by applying various negative pressures on the axonal membrane with a high-speed pressure-clamp device under the cell-attached configuration (Fig. 1B). The single-channel conductance was approximately 90 pS at 80 mV, which was consistent with the channel conductance of the heteromeric TREK-1/TRAAK channels [4] (Fig. 1C). The single-channel conductance was not affected by membrane stretch (Fig. 1C). The single-channel event numbers and open probability increased in a pressure-dependent manner and showed a significant difference from the negative pressure of 60 mmHg or more (Fig. 1D, E). These results show that TREK-1/TRAAK channels are highly sensitive to mechanical membrane stretch, and suggest that TREK-1/TRAAK activation by membrane stretch may modulate neuronal excitability at the NRs.

At NRs, strong leak potassium outward (lK_leak_) currents following depolarizing voltage steps mainly mediated by activation of TREK-1/TRAAK channels [4]. To determine whether membrane stretch enhances the IK_leak_ currents by this activation at the NRs, we applied positive pressure into cells through a recording pipette under the whole-cell configuration (Fig. 1F). By applying positive pressure in this configuration, we applied membrane tension in the same direction from inside to outside of the cell as cell-attached configuration. Because the seal between the cell membrane and patch pipette is easily broken by internal positive pressure, we applied a maximum pressure of 10 mmHg for the experiment. This positive pressure enhanced the outward currents in response to the voltage steps (Fig. 1F). Consistent with single-channel activities, intra-pipette positive pressure significantly increased IK_leak_ conductance compared to that at 0 mmHg (Fig. 1G). To confirm whether the enhanced outward currents were mediated by TREK-1/TRAAK channels, we pharmacologically blocked these channels by applying Ba^2+^. The bath application of Ba^2+^ significantly suppressed pressure-enhanced outward currents and IK_leak_ conductance (Fig. 1G, H). These results clearly show that intra-pipette pressure enhances K + conductance by activating TREK-1/TRAAK channels at the NRs.

We further confirmed whether membrane stretch modulates nodal membrane excitability in NRs in the current-clamp mode. The input resistance was significantly decreased by 10 mmHg intra-pipette positive pressure (Fig. 1I, J). The resting membrane potential was significantly decreased (Fig. 1K) and the rheobase was significantly increased by an intra-pipette pressure of 10 mmHg (Fig. 1L). The blockage of TREK-1/TRAAK channels by Ba^2+^ reversed the changes in the intrinsic electrophysiological properties induced by intra-pipette pressure (Fig. 1J–L).

In conclusion, our study demonstrates that mechanosensitive TREK-1/TRAAK channels may be activated by axonal stretch. The activation could suppress neuronal excitability. We previously found that TREK-1 and TRAAK are highly expressed in the NRs of mammalian sensory nerves, which leads to AP repolarization instead of voltage-gated K^+^ channels [4]. In the present study, single-channel activities with channel conductance of 90 pS (TREK-1/TRAAK-like) were increased by membrane stretch in a pressure-dependent manner. Consistent with single-channel activity, intra-pipette positive pressure increased IK_leak_ currents and suppressed membrane excitability. Furthermore, pharmacological blockage of TREK-1/TRAAK channels reversed the changes in intrinsic electrophysiological properties induced by intra-pipette pressure. These results indicate the importance of TREK-1/TRAAK channels in the prevention of ectopic AP discharge at the axon by intense mechanical nerve stretch under physiological conditions.

As a limitation of this study, we used intra-pipette pressure to study ion channel function at the NRs instead of axonal stretch, because the membrane seal for patch-clamp recording is easily disrupted by membrane displacement. Thus, the application of mechanical pressure with a high-speed pressure-clamp device may not fully represent axonal stretch.

In conclusion, our study demonstrates the effect of membrane stretching on the intrinsic electrophysiological properties of NR. The findings provide important insights into the pathology of diseases, such as demyelination.

## Supplementary Information

**Additional file 1.** Materials and methods.

## Data Availability

All data generated during this study are available from corresponding author on reasonable request (Additional file 1).

## Abbreviations

AP: Action potential
K2P channel: Two tandem pore domain potassium channel
lK_leak_: Leak potassium outward
NRs: Nodes of Ranvier
TRAAK: TWIK-related arachidonic acid-activated K^+^ channel
TREK-1: TWIK-related K^+^ channel
