# Supplementary material for: Axonal membrane stretch suppresses neuronal excitability by activating mechanosensitive K2P channels at the node of Ranvier
Source: Mol Brain. 2023 Jan 17;16:8. doi: 10.1186/s13041-023-01000-6 (PMC9843873; doi:10.1186/s13041-023-01000-6)
Supplement: Supplementary file 1 — Additional file 1. Materials and methods. [file 13041_2023_1000_MOESM1_ESM.docx]

**Materials and Methods (Additional)**

*Animals.* Experiments were performed on 7-10 week old male Sprague Dawley rats (SLC Japan). Rats were housed in standard cages with *ad libitum* access to food and water in a temperature-controlled room (23°C) and maintained on a 12-h light/dark cycle. All procedures were approved by Hyogo University of Health Sciences Committee on Animal Research. All experimental procedures were performed in accordance with NIH guidelines for the care and use of laboratory animals.

*Ex vivo sciatic nerve preparation.* The rats were euthanized by an overdose of isoflurane, and sciatic nerve bundles were harvested and kept in ice-cold Leibovitz’s L-15 medium (Gibco; Thermo Fisher Scientific). Connective tissues surrounding nerve bundles were removed with fine forceps, and the sample was affixed in a recording chamber using a tissue anchor and submerged in a normal Krebs solution that contained (in mM):117 NaCl, 3.5 KCl, 2.5 CaCl_2_, 1.2 MgCl_2_, 1.2 NaH_2_PO_4_, 25 NaHCO_3_, and 11 glucose. Krebs solution was saturated with 95% O_2_ and 5% CO_2_, had a pH of 7.35, an osmolarity of 324 mOsm at a room temperature of 24°C. Sciatic nerve bundles were briefly exposed to a mixture of 0.07% dispase (type II, Godo Shusei) and 0.07% collagenase (Nacalai Tesque) in Krebs solution for 5 min at the same room temperature.

*Pressure-clamped patch-clamp recordings at NR.* The procedures were performed as previously described [1]. NRs in myelinated nerves of ex vivo trigeminal nerve preparations were visualized under a 40× water immersion objective using an infrared CCD camera (IR-1000, DAGE-MTI). Patch-clamp recordings were applied to the NR of the sciatic Aβ-afferent nerve fibers (8 to 10 μm including myelin thickness). After filling the recording electrode internal solutions, the electrode resistance was 8 MΩ. For all experiments, the recording electrode was filled with an internal solution containing the following (in mM):105 K-gluconate, 30 KCl, 0.5 CaCl_2_, 2.4 MgCl_2_, 5 EGTA, 10 HEPES, 5 Na_2_ATP, and 0.33 GTP-TRIS salt. The pH of the solution was adjusted to 7.35 with KOH. The recording electrode was connected to a high-speed pressure-clamp device (HSPC-1; ALA Scientific Instruments) to control its internal pressure. Signals of voltage-clamp experiments were recorded and amplified using an Axopach 200B amplifier, filtered at 2 kHz, and sampled at 10 kHz using pCLAMP 10 software (Molecular Devices). Signals of current-clamp recordings for APs at the NR were low-pass filtered at 2 kHz, and sampled at 50 kHz.

*Single-channel recording.* To record single-channel activity, recordings were performed on axonal membranes at NRs in a cell-attached configuration. After forming a gigaohm seal, the intra-electrode pressure was adjusted to 0 mmHg using a high-speed pressure-clamp device. Single-channel recordings were performed using a K^+^-base recording electrode internal solution with a K^+^ concentration of 135 mM (reversal potentials of K^+^ were near 0 mV). The single-channel activities were recorded at 80 mV and the voltages in the recording electrodes were in reference to the resting membrane potentials of −80 mV at the NRs. The effects of membrane stretch on single-channel activity were determined by applying stepwise negative pressure using a patch-clamp recording pipette. Stepwise pressure (from 0 to -100 mmHg) was generated using a high-speed pressure-clamp device.

*Testing electrophysiological properties and neuronal excitability.* To determine the intrinsic electrophysiological properties, including both passive and active properties of nodal membranes, patch-clamp recordings were performed under a whole-cell current-clamp configuration. Step-current pulses were injected into the NR through a recording electrode. Step currents ranged from −100 to 1800 pA in increments of 50 pA per step, and the duration of each step was 1 s. The voltage steps were applied from −102 mV to +58 mV (voltage command of −90 to +70 mV) with increments of 10 mV for each step and a step duration of 500 ms. Unless otherwise indicated, the membrane potentials mentioned in the text were corrected for the calculated junction potential of 12 mV.

*Drug application.* The drug was delivered to the cells from a short tube (0.2 cm L, 500 μm internal diameter) with the outlet 2 mm away from the recorded cells. Ba^2+^ (5 mM) was applied through the same tube.

*Data analysis.* Single-channel activities, intrinsic electrophysiological properties, and voltage-activated currents were analyzed using the Clampfit 10 software. Unless otherwise indicated, all data are reported as the mean ± SEM. Statistical comparisons were made using GraphPad Prism 9 software with *p < 0.05, **p < 0.01, and ***p < 0.001, and one-way ANOVA with Tukey's multiple comparison test.

Reference

1. Kanda H, Tonomura S, Dai Y, Gu JG. Protocol for pressure-clamped patch-clamp recording at the node of Ranvier of rat myelinated nerves. STAR Protoc [Internet]. Elsevier; 2021;2:100266. Available from: http://dx.doi.org/10.1016/j.xpro.2020.100266
